# Sexual characteristics of patients with prostate cancer seen for radiation treatment

**DOI:** 10.3332/ecancer.2023.1577

**Published:** 2023-07-20

**Authors:** Abbas Adus-salam, Mutiu Jimoh, Chiamaka Godsgift Ehiedu

**Affiliations:** 1 Department of Radiation Oncology, University College Hospital/University of Ibadan, Ibadan 200212, Nigeria; 2 University College Hospital, Ibadan 200005, Nigeria

**Keywords:** sexual characteristics, prostate cancer, sexual dysfunction

## Abstract

**Background:**

Prostate cancer was estimated to be the second most diagnosed cancer and the fifth leading cause of cancer mortality among men, with an estimated 1.4 million new cases and 375,000 deaths globally in 2020. There are significant changes in sexual activities and subsequent changes in quality of life associated with the diagnosis and treatment of prostate cancer. Sexual problems experienced by prostate cancer patients include erectile dysfunction, reduced sexual desire, reduced sexual function, problems with ejaculation, as well as problems with orgasm, and these could occur before and/or after treatment. This study aims to highlight the sexual characteristics of prostate cancer patients, which would help identify altered sexuality that might require intervention by healthcare providers.

**Method:**

All patients who presented with pathologically diagnosed, organ-confined prostate cancer referred for high-dose-rate brachytherapy were approached for participation in the study. An interviewer-administered questionnaire was administered to the consenting patients.

**Results:**

A total of 56 patients gave consent for the study out of 60. All the patients were married, with 5 (8.9%) having multiple wives. Only ten respondents (17.9%) reported having other sexual partners besides their wives. More than half of the patients (34) (60.7%) started having sexual intercourse between the ages of 18 and 20. Many patients claimed that the diagnosis of prostate cancer had affected their sexual lives. About half of the respondents (44.6%) believed that their partners were less satisfied with their sexual performance, as evidenced by the loss of partners (5.4%), partners refusing sexual advances (14.3%), partners complaints (10.7%), and partners' reduced inclination to ask for sex (33.9%). One patient expressed fears of passing the disease to their partners.

**Conclusion:**

The management of prostate cancer should include sex therapy and rehabilitation in couples from the point of diagnosis to maintain sexual function as close as possible to that in the general population in order to maintain an improved quality of life.

## Introduction

Prostate cancer was estimated to be the second most diagnosed cancer and the fifth leading cause of cancer mortality among men, with an estimated 1.4 million new cases and 375,000 deaths globally in 2020 [1]. In Africa and Nigeria, prostate cancer was estimated to be the most commonly diagnosed malignancy and the leading cause of cancer mortality among men in 2020, with an estimated 93,173 new cases and 47,249 mortalities in Africa and 15,306 new cases and 8,517 mortalities in Nigeria [2, 3]. Nigeria accounted for 16.4% of the new prostate cancer cases and 18% of prostate cancer mortalities in Africa in 2020 [2, 3].

Incidence rates of prostate cancer vary across the world, with the lowest rates in Asia and Northern parts of Africa and the highest rates in The Caribbean, Europe, Australia, New Zealand, Northern America and Southern Africa, while prostate cancer mortality rates are highest in The Caribbean, Sub-Saharan Africa and Micronesia/Polynesia [1]. Some identified risk factors for prostate cancer include increasing age, genetic mutations, family history [1], blacks [4], smoking and being overweight [5].

There are significant changes in sexual activities and subsequent changes in quality of life associated with the diagnosis and treatment of prostate cancer [6]. Sexual problems experienced by prostate cancer patients include erectile dysfunction, reduced sexual desires, reduced sexual function, problems with ejaculation, as well as problems with orgasm, and these could occur before and/or after treatment [7–11]. Sexual dysfunction in prostate cancer patients could result from prostate cancer, prostate cancer treatments, and psychiatric disorders (such as depression, fear, anxiety and psychologic instability) resulting from prostate cancer diagnosis or its treatment [12–14].

Erectile dysfunction is the most extensively studied sexual dysfunction [15] and is defined as consistent or recurrent inability to attain and/or maintain penile erection sufficient for sexual activity by a man for ≥3 months [16]. Some studies have shown erectile dysfunction incidence post-radical prostatectomy to be between 20% and 90% [17–19]. Another study revealed the incidence of impotence in previously potent men as 65% after non-nerve-sparing prostatectomy, 58.6% after unilateral nerve-sparing prostatectomy, and 56.0% after bilateral nerve-sparing prostatectomy [20]. The incidence of erectile dysfunction after radiotherapy was reported to be 19%, 28%, 26% and 40% before radiotherapy, on the last day of radiotherapy, 2 months after radiotherapy, and 16 months after radiotherapy, respectively, with radiotherapy dose of 70.2–72Gy [21]. Following high-dose-rate brachytherapy, the incidence of erectile dysfunction was reported to be between 10% and 51%, with a median of 31.5% [22]. A meta-analysis that assessed the rate of erectile dysfunction after treatment for localised prostate cancer revealed the predicted probability of erectile dysfunction after high-dose-rate brachytherapy, external beam radiotherapy, high-dose-rate brachytherapy and external beam radiotherapy, nerve-sparing prostatectomy, radical prostatectomy, and cryotherapy as 24%, 45%, 40%, 66%, 75% and 87%, respectively [23]. Other factors associated with erectile dysfunction include increasing age, hypertension, diabetes mellitus and medications [24–26]. Changes in sexuality affect not only males with prostate cancer but also their partners [27] as both patients and their female partners suffer psychological effects of the diagnosis and treatment of prostate cancer [12, 28, 29].

Preservation of sexual function is important in the management of patients with localised prostate cancer [12] and health professionals need to keep this in mind to ensure better quality of life for prostate cancer patients and their partners [30]. This study aims to highlight the sexual characteristics of prostate cancer patients, which would help identify altered sexuality that might require intervention by healthcare providers. Also, this study will help fill some knowledge gaps, as studies on the sexuality of prostate cancer patients in Nigeria are sparse.

## Aims and objectives

The aim of the study is to report on the sexual characteristics of the patients who presented with pathologically diagnosed prostate cancer.

## Method

This study is a descriptive cross-sectional study carried out amongst pathologically confirmed, localised prostate cancer patients presenting at the Department of Radiation Oncology, University College Hospital, Ibadan, for high dose rate prostate brachytherapy between August 2020 and April 2022. The study questionnaire (Appendix 1) is made up of two sections administered to study participants with the help of a research assistant who helped in filling and translating medical terms in the study questionnaire for the ease of understanding of participants. All 60 patients seen within this period were approached for the study. However, only 56 patients gave written informed consent for the study. Data were collected from consenting patients with the aid of a questionnaire administered by the researchers. Other necessary clinical information was obtained from the patient’s case notes. The collected data was analysed using SPSS v. 22, and the results were presented in tables and frequencies.

## Results

**Figure 1.**
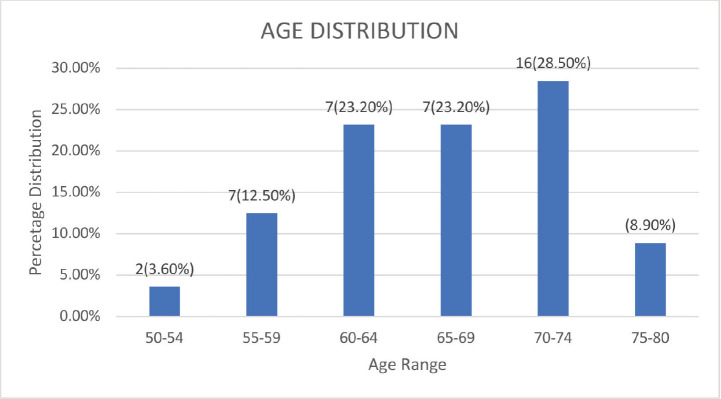
Age distribution of patients.

A total of 56 patients gave consent for the study out of 60 patients seen during the period. Their ages ranged from 51 to 79, with a mean age of 66.45 years and a SD of 6.785 (Figure 1). All patients had localised adenocarcinoma of the prostate, with prostate volume ranging between 23 and 96 mLs and prostate-specific antigen (PSA) ranging between 5.8 and 124.5 ng/mL, and were radiation treatment naïve. They all subsequently high dose brachytherapy 27 Gy in 2#. All the patients were married, with 5 (8.9%) having multiple wives. Only ten respondents (17.9%) reported having other sexual partners besides their wives. More than half of the patients (34%, or 60.7%) started having sexual intercourse between the ages of 18 and 20 years. The earliest was 15 (1 patient) years, while 37 years was the most advanced age for first sexual intercourse.

Most of the patients (91.1%) were sexually active prior to the diagnosis of prostate cancer. Only five patients (8.9%) were not sexually active before the diagnosis. Those who are not sexually active increased to 37.5% following the diagnosis (Table 1). Prior to diagnosis, most patients had sex once (20 patients, 35.7%) or twice (22 patients, 39.3%) per week.

Only a few patients (7, 12.5%) reported using sex-enhancing substances like drugs and alcohol. Sildenafil and alcohol were the most popular substances used. Only three patients (5.4%) reported previous oral sex. Another 6 (10.7%) had a previous history of sexually transmitted diseases (STDs), some on multiple occasions. Gonorrhoea was the most commonly diagnosed STD. Others are staphylococcal and herpes infections. There was no history of syphilis among the patients.

Most of the patients knew their HIV status and that of their partners, and they all reported being HIV-negative.

Many patients claimed that the diagnosis of prostate cancer had affected their sexual lives. About half of the respondents (44.6%) believed that their partners were less satisfied with their sexual performance, as evidenced by the loss of partners (5.8%), partners refusing sexual advances (14.3%), partners' complaints (10.7%), and reduced inclination of the patients’ partners for sex (33.9%) (Table 1). One patient expressed fears of passing the disease to their partners.

## Discussion

This study showed that the mean age of patients with prostate cancer presenting for brachytherapy was 66.45 years (SD: 6.785 years) and the ages ranged from 51 to 79 years. This finding is consistent with those from various studies globally and locally [1, 31–37]. This could be due to the increasing incidence of prostate cancer with increasing age [38]. The diagnosis and treatment of prostate cancer in this age group could constitute additional morbidity, as patients within this age group are at increased risk of having other co-morbidities.

All patients in this study were married. This is similar to the findings of a study carried out in Lagos and another comparative study of clinicopathologic features between Nigerian patients in Abuja and South African patients with prostate cancer. In the Lagos study, all patients were either married (95.1%), separated (2.1%), or widowed (2.8%), while all patients from Nigeria in the comparative study were married [36, 37]. This could be due to sociocultural practices among Nigerians, as the proportion of married men increases with increasing age, as demonstrated by the 2018 national demographic health survey, which showed that over 90% of men between the ages of 40 and 49 were married [39].

Less than a tenth (8.9%) of the patients had multiple wives. The finding is slightly different from that of a study carried out amongst prostate cancer patients in Sokoto State, Nigeria, which showed that about 28.6% of patients were married in a polygamous setting [40]. This could be due to the predominance of Muslims in the Northern part of Nigeria, whose religion permits polygamy with a maximum of four wives [40]. Possible reasons for the preference for monogamy include religious, social and economic reasons [41].

In this study, 17.9% of the patients had sexual partners apart from their wife/wives. This finding is similar to the findings of a case-control study carried out in the United States of America, which showed that 27% of patients with prostate cancer have had sexual intercourse with prostitutes [42]. Extramarital affairs have been shown to have a significant association with younger age, urban residential areas, Yoruba ethnicity, early age of sexual debut, monogamy and higher levels of education [39, 43] and some of these factors could account for the findings in our study.

About three-fifths of the patients had their sexual debut between the ages of 18 and 20, with 15 years being the earliest and 37 years the latest. This finding is in tandem with studies carried out in the United States of America and Turkey, which demonstrated the age of first sexual intercourse among patients with prostate cancer to be 16–19 and 18 ± 6 years, respectively [42, 44]. This could be due to the existing sociocultural practices, as the median age of coitarche in the general population amongst men was revealed to be 21.7 years by the national demographic health survey in Nigeria [39]. Most pre-diagnosis sexual characteristics of patients in this study are almost similar to those of the general population, as shown by the national demographic health survey, and efforts should be made by the healthcare providers to ensure that the changes after diagnosis and treatment are as minimal as possible to ensure optimal quality of life.

The majority (91.1%) of the patients in this study were sexually active prior to diagnosis, and this proportion decreased to 62.5% after diagnosis. Findings similar to this were also made by Albaugh *et al* [45] who reported that the majority (74%) of the prostate cancer patients did not report erectile dysfunction prior to the diagnosis of prostate cancer while another study reported that 58.7% of patients were sexually active post-treatment [46]. The reduction in sexual activity could be due to factors such as the psychological trauma of diagnosis in both patients and partners, treatment, and the psychological stress of treatment [12–14, 28, 29]. Such a decrease in sexual activity can lead to decreased sexual satisfaction and a subsequent decrease in overall quality of life.

About 75% of the patients who were sexually active in this study had sexual intercourse at least once or twice per week, which is in tandem with the study carried out by Rousseau *et al* [47] where 80% of the prostate cancer patients had at least one sexual intercourse per week. The finding from this study is also similar to that carried out in the United States of America, which revealed that more than half of the patients with prostate cancer had sexual intercourse about 1–2 times per week [42].

Sex stimulants such as drugs (Sildenafil) and alcohol were used by patients (12.5%) in this study. Phosphodiesterase five inhibitors are part of the treatment options for the management of erectile dysfunction, with an 81% response rate following their use in prostate cancer patients with erectile dysfunction [48]. Alcohol, on the other hand, is seen by many as sex-enhancing, and this is ascribed to its disinhibitory properties [49]. Compared to the proportion of patients with reduced sexual activity, the proportion of patients that used sex enhancers was low, and this could be due to fear of drug interaction with treatment or under-reporting as a result of privacy concerns.

Three patients (5.4%) reported a prior history of oral sex. There is a dearth of literature relating to oral cancers in heterosexual prostate cancer patients. However, a study carried out among men who have sex with men revealed that oral sex was the most frequent form of sexual practice after masturbation [50]. Socio-cultural norms or the unpopularity of oral sex in Nigeria could account for the underreporting of oral sex practices in Nigeria. While there is no literature reporting an association between oral sex and prostate cancer, oral sex has been recommended as a form of sexual activity that can provide sexual pleasure and an avenue to build intimacy amongst partners [51].

Gonorrhoea was the most commonly reported sexually transmitted infection (STI) among 10.7% of patients who had a prior history of STIs. This is similar to a Mexican study that showed that 16.6% of the men reported previous STI, with gonorrhoea being the most common, and that a previous history of gonorrhoea was associated with an increased risk of prostate cancer [52]. The findings in this study were also in tandem with those from the case-control study done in the United States of America, which revealed that about 13% of patients with prostate cancer had a history of previous gonorrhoea infections [42]. Some studies have reported that age at first intercourse, number of sexual partners, and history of gonorrhoea may play a role in the aetiopathogenesis of prostate cancer, while others have demonstrated no association between the above sexual characteristics and prostate cancer [40, 42, 44, 53].

The diagnosis of prostate cancer was reported to affect the sexual lives of most patients. This could be due to psychological stress, fear and anxiety, resulting in altered sexual lives [12–14]. The diagnosis and treatment of prostate cancer can also affect the sexuality of patients’ partners [12, 29–54], and in our study, 44.6% of patients believed that their partners were less satisfied sexually, which led to the reduced inclination for sex by patients partners (33.9%), refusal of sexual advances by patients partners (14.3%), complaints by patients partners (10.7%), and loss of a partner (5.4%). This brings to the fore a need by partners of patients with prostate cancer that needs to be catered for. Significant to note is that one patient’s sexual life was altered due to fear of transmitting prostate cancer to his partner. This highlights the need for proper patient education to prevent altered sexual function and a subsequent decrease in quality of life due to a lack of information.

This study is the first, to the best of our knowledge, to evaluate the sexual characteristics of treatment-naive prostate cancer patients with localised disease in Nigeria. It is, however, limited by the sample size. Further analytical studies with a larger sample size need to be carried out to assess all aspects of sexual dysfunction in prostate cancer patients as well as their partners for prompt pre-emptive management to ensure improved quality of life for prostate cancer patients and their partners.

## Conclusion

Prostate cancer is a disease of public health importance due to its burden on society. The diagnosis and treatment of prostate cancer have a significant impact on the sexuality of prostate cancer patients and their partners, with a subsequent impact on their quality of life. Thus, the management of prostate cancer should include sex therapy and rehabilitation in couples from the point of diagnosis to maintain sexual function as close as possible to that in the general population in order to maintain an improved quality of life.

## Conflicts of interest

The authors declare no competing interests.

## Funding

None.

## Consent for publication

All authors agreed on the publication of this manuscript.

## Informed consent

Informed consent was obtained from all participants and/or their legal guardians.

## Ethical consideration

All procedures performed in studies involving human participants were in accordance with the ethical standards of the institutional and/or national research committee and with the 1964 Helsinki Declaration and its later amendments or comparable ethical standards. The study was approved by the Bioethics Committee of the University College Hospital Ibadan.

## Availability of data and materials

The datasets generated during and/or analysed during the current study are available from the corresponding author on reasonable request.

## Figures and Tables

**Table 1.** Occupation, Gleason score, sexual characteristics and sexual impact of prostate cancer diagnosis on patients.

Occupation	Frequency (%)
Retired	30(53.6)
Trading	6(10.7)
Medical doctor	5(8.9)
Lecturer	4(7.1)
Artisan	4(7.1)
Businessman	4(7.1)
Engineer	3(5.4)
	
**Gleason score**	**Frequency (%)**
3 + 3	24(42.8)
3 + 4	20(35.7)
4 + 3	5(8.9)
5 + 3	2(3.6)
5 + 4	3(5.4)
5 + 5	2(3.6)
	
**Sexual characteristics**	**Frequency (%)**
Married	56(100)
Polygamous	5(8.9)
Other sexual partners	10(17.9)
Sexually active before diagnosis	51(91.1)
Sexually active after diagnosis	35(62.5)
Use of sexual stimulants	7(12.5)
Previous oral sex	3(5.4)
Previous STD	6(10.7)
	
**Impact of prostate cancer diagnosis**	**Frequency (%)**
Partner less satisfied	25(44.6)
Decreased inclination for sex by partners	19(33.9)
Partners' refusal of sexual advances	8(14.3)
Complains about sex by partners	6(10.7)
Partner left	3(5.4)

## Appendix 1. Questionnaire.

**Section A: Sociodemographic data**

**Study center: ____________________ Year of study ___________________**

Age:______ Religion:____________ Tribe:____________ State (Origin):____________ State (Residencial)____________ Occupation: ____________

Marital Status: Single ( ) Married ( ) Separated ( ) Divorced ( ) Widow ( ) Co-habiting( ) Others

**Section B: Clinical characteristics**

**Past history** Hypertension ( ) Diabetes ( ) Seizures ( ) Asthma ( )

Others (specify) ___________________Drug history: _____________________________ Previous surgeries: _______________________________

**PSA:**

**Histologic type:___________________**

**Perineural invasion:** Yes ( ) No ( )**Lymphovascular invasion** Yes ( ) No ( )

**Gleason score**

**Grade**

**Stage**

**Nature of specimen:** Transrectal ( ) Transurethral ( ) Transperineal ( )

**Prior hormone therapy and duration**.

**Prior EBRT** (Dose, Fractionation, Dose Per Fraction Duration and Facility).

**Prior chemotherapy (regimen and duration)**

**Brachytherapy** (Dose, Fractionation, Dose/Fraction and Duration)

**EBRT after brachytherapy** (Dose, Fractionation, Dose/Fraction, Duration, Facility)

**Section B: Sexual characteristics**

Multiple wives: Yes ( ) No ( ) No of wives: ____________

Other sexual partners: Yes ( ) No ( ) No of sexual partners: ________

Age at first sexual intercourse? __________

Do you consider yourself sexually active before this diagnosis?Yes ( ) No ( )

Do you consider yourself sexually active after this diagnosis?Yes ( ) No ( )

How often do you have sex in 1 week/month: ____/Week or ____/Month

Have you ever used sexual aid like drug/alcohol?

List them: ______________________________________________

Have you ever had oral sex performed on you before? Yes( ) No( ) How often?____

Previous history of sexually transmitted disease? Yes( )No ( ) How many times?____

Do you know the diagnosis? Yes( ) No( ) List___________________

Do you know your HIV status? Yes( ) No( ) Negative or Positive _________

If positive, are you on treatment? Yes ( ) No ( )

Do you know your partners’ HIV status?Yes ( ) No ( ). Negative ( ) Positive ( )

Do you think your partner(s) are often satisfied with your performance?

Partner(s) left ( ),

Partner(s) less inclined to ask for sex ( ),

I am less inclined to ask for sex ( ),

My performance has diminished ( ),

Partner(s) complaining ( ),

Partner(s) and I have fears of passing on the disease ( )
